# Antibacterial and chemical profiling of *Ulva intestinalis* collected from Egypt

**DOI:** 10.1038/s41598-025-27913-x

**Published:** 2025-11-29

**Authors:** Dina Hussien Ibrahim, Hoida Ali Badr, Magdy A. Abu-Gharbia, Aly E. Abo-Amer

**Affiliations:** Department of Botany and Microbiology, Faculty of Science, Sohag University, Sohag, 82524 Egypt

**Keywords:** Antimicrobial activity, Seaweeds, Antibiofilm activity, Gas Chromatography–Mass spectrometry, FTIR analysis. docking and molecular dynamics, Biochemistry, Biotechnology, Drug discovery, Microbiology

## Abstract

The increasing limitations of antibiotic therapy, due to adverse effects and the alarming rise of bacterial resistance, underscore the urgent need for novel antimicrobial alternatives. This study investigated the antibacterial and antibiofilm activities of crude extracts from the green seaweed *Ulva intestinalis* as potential natural substitutes for conventional antibiotics. Samples were collected from the Mediterranean coast of Alexandria, Egypt, and extracted using ethanol, methanol, and acetone. The antibacterial activity against *Staphylococcus aureus* was assessed via the agar well diffusion method. Among the tested extracts, the ethanol extract at 200 µg/mL showed the largest inhibition zone (35.03 ± 0.04 mm), whereas the acetone extract exhibited the lowest activity (15.7 ± 0.09 mm) at the same concentration. The antibiofilm efficacy, evaluated using a 96-well microtiter plate assay, revealed that the methanolic extract inhibited biofilm formation by 91.26% at 200 µg/mL, the highest tested concentration. GC–MS analysis identified several bioactive constituents, including pentadecane, eicosane, and 2,6,10,14-tetramethylpentadecane, whereas FTIR spectra confirmed the presence of phenolic, alcoholic, aliphatic, ketonic, aromatic, and amine functional groups. Molecular docking studies demonstrated strong binding affinities of selected compounds toward key *S. aureus* targets (RNA polymerase, DNA gyrase, and PBP2a), suggesting potential mechanisms involving disruption of transcription, DNA replication, and β-lactam resistance. Overall, these results highlight *U. intestinalis* as a promising marine-derived source of antimicrobial and antibiofilm agents with potential pharmaceutical applications.

## Introduction

 The global rise in antibiotic-resistant pathogens poses a severe threat to public health, undermining the effectiveness of existing antimicrobial therapies and complicating the management of infectious diseases. Among these pathogens, *Staphylococcus aureus* has emerged as one of the most prevalent causative agents of both hospital- and community-acquired infections. Its remarkable ability to form biofilms a complex microbial structure that provides protection against antibiotics and host immune defenses further enhances its persistence and pathogenicity^1,2^. Consequently, infections caused by biofilm-forming *S. aureus* are difficult to eradicate and often lead to chronic or recurrent conditions. In light of this challenge, there has been growing scientific interest in exploring natural products, particularly those derived from marine organisms, as alternative sources of novel antimicrobial agents^3,4^. Marine algae, commonly referred to as seaweeds, represent a rich reservoir of bioactive compounds with diverse pharmacological properties, including antibacterial, antifungal, antioxidant, and anti-inflammatory effects^5,6^. Among them, green seaweeds of the genus *Ulva* (Chlorophyta) have attracted special attention due to their abundance, ecological significance, and high content of biologically active metabolites such as polyphenols, flavonoids, alkaloids, terpenoids, and sulfated polysaccharides^7^. These metabolites are known to interfere with bacterial cell wall integrity, membrane permeability, quorum sensing, and biofilm formation, making *Ulva* species potential candidates for antimicrobial drug discovery. This highlights the urgent need to explore alternative antimicrobial agents from marine sources, such as *U. intestinalis*, to address the growing threat of drug-resistant pathogens. Previous studies have reported the antibacterial potential of *Ulva* extracts against a variety of Gram-positive and Gram-negative bacteria^8^. However, most existing research has been limited to preliminary screenings, often lacking detailed chemical characterization or mechanistic interpretation of the observed bioactivity. Furthermore, the antimicrobial potential of *Ulva intestinalis* collected from the Egyptian Mediterranean coast remains underexplored, despite the influence of regional environmental factors on the composition and potency of algal metabolites. Therefore, the present study aims to comprehensively evaluate the antibacterial and antibiofilm activities of crude extracts of *Ulva intestinalis* against *Staphylococcus aureus*, utilizing different solvent extraction systems (ethanol, methanol, and acetone). In addition, phytochemical profiling using GC–MS and FTIR analyses was conducted to identify the major bioactive constituents. Molecular docking was further employed to predict potential interactions between identified compounds and essential bacterial target proteins^9^. The findings of this study may contribute to the development of marine-derived antimicrobial agents capable of combating drug-resistant and biofilm-forming pathogens. Although the antimicrobial potential of *Ulva intestinalis* has been previously reported from different geographical regions, the present study provides a novel contribution by investigating samples collected from the Mediterranean coast of Egypt, where environmental factors such as salinity, temperature, and nutrient availability influence the algal metabolite profile. This study integrates biological assays with chemical and computational analyses to establish a comprehensive link between the phytochemical composition and the antimicrobial mechanisms of *U. intestinalis*. Such an integrative approach, supported by GC–MS, FTIR, and molecular docking, offers deeper mechanistic insights into how algal-derived compounds may inhibit both planktonic and biofilm-associated *Staphylococcus aureus*. Hence, this work not only enriches the regional understanding of Egyptian marine biodiversity but also broadens the global pharmacological relevance of *U. intestinalis* as a promising source of natural antimicrobial agents.

## Results

### Antibacterial activity by the agar well diffusion method

All extracts of *Ulva intestinalis* (methanolic, ethanolic, and acetone) showed significant antibacterial activity against *Staphylococcus aureus*. The ethanolic extract exhibited the highest inhibition zone (35.03 ± 0.04 mm) at 200 µg/mL, followed by the methanolic extract (33.03 ± 0.24 mm). Both extracts were significantly more effective than the positive control (19.9 ± 0.08 mm) shown in Table 1. The acetone extract showed moderate activity, with an inhibition zone of 15.7 ± 0.09 mm, close to the control value.

**Table 1:** Antibacterial activity of *Ulva intestinalis* extracts against *Staphylococcus aureus*. Data are presented as mean ± SD of triplicate measurements (*n* = 3). N/A: no activity observed. Values indicate a clear concentration-dependent increase in antibacterial activity.

Solvent	Concentration (µg/ml)	Mean diameter of inhibition zone (mm) ± SD
Methanol	50	15.03 ± 0.16
	100	25.53 ± 0.16
	**200**	**33.03 ± 0.24**
**Ethanol**	**50**	**11.7 ± 0.16**
	**100**	**25.36 ± 0.09**
	**200**	**35.03 ± 0.04**
**Acetone**	**50**	**N/A**
	**100**	**5.9 ± 0.08**
	**200**	**15.7 ± 0.09**
**Negative control (DMSO)**	**—**	**N/A**
**Positive control (Gentamicin**,** 10 µg)**	**—**	**19.9 ± 0.08**

### Antibiofilm activity

**Fig. 1:**
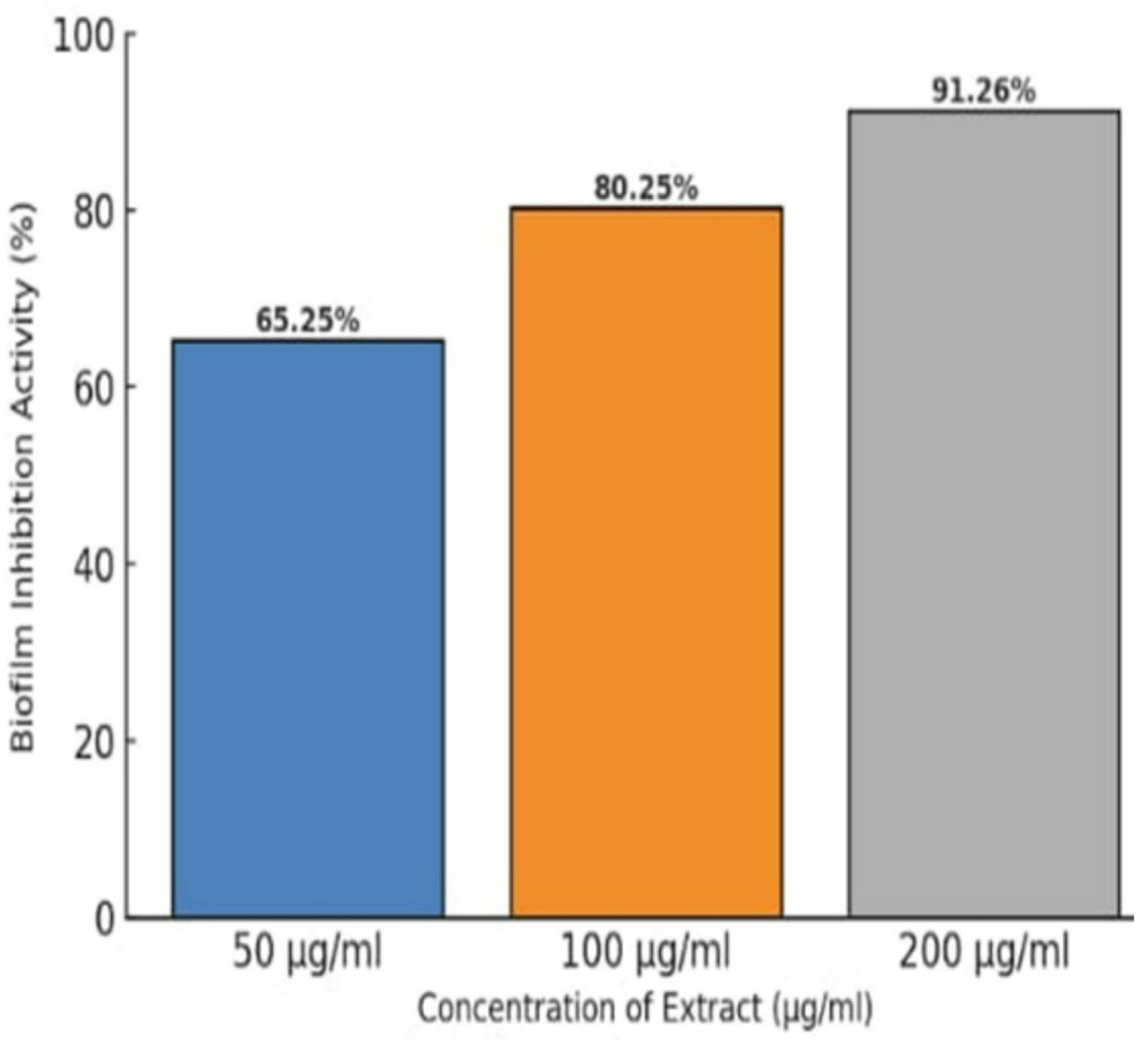
Percentage of biofilm inhibition of *Staphylococcus aureus* treated with *Ulva intestinalis* methanolic extract at various concentrations (50–200 µg/mL). Values represent mean ± SD (*n* = 3, *p* < 0.05).

At a concentration of 200 µg/mL, the antibiofilm formation inhibitory activity of *Ulva intestinalis* extract against *Staphylococcus aureus* was evaluated. The extract exhibited a biofilm inhibition rate of 91.26%, demonstrating a significant ability to prevent biofilm formation, as shown in Fig. 1. These findings indicate that the extract effectively suppresses the initial stages of biofilm development under the tested conditions. Overall, the results highlight the potential of *U. intestinalis* as a natural antibiofilm agent for inhibiting biofilm formation associated with *S. aureus*.

### Gas chromatography–mass spectrometry (GC–MS)

**Fig. 2:**
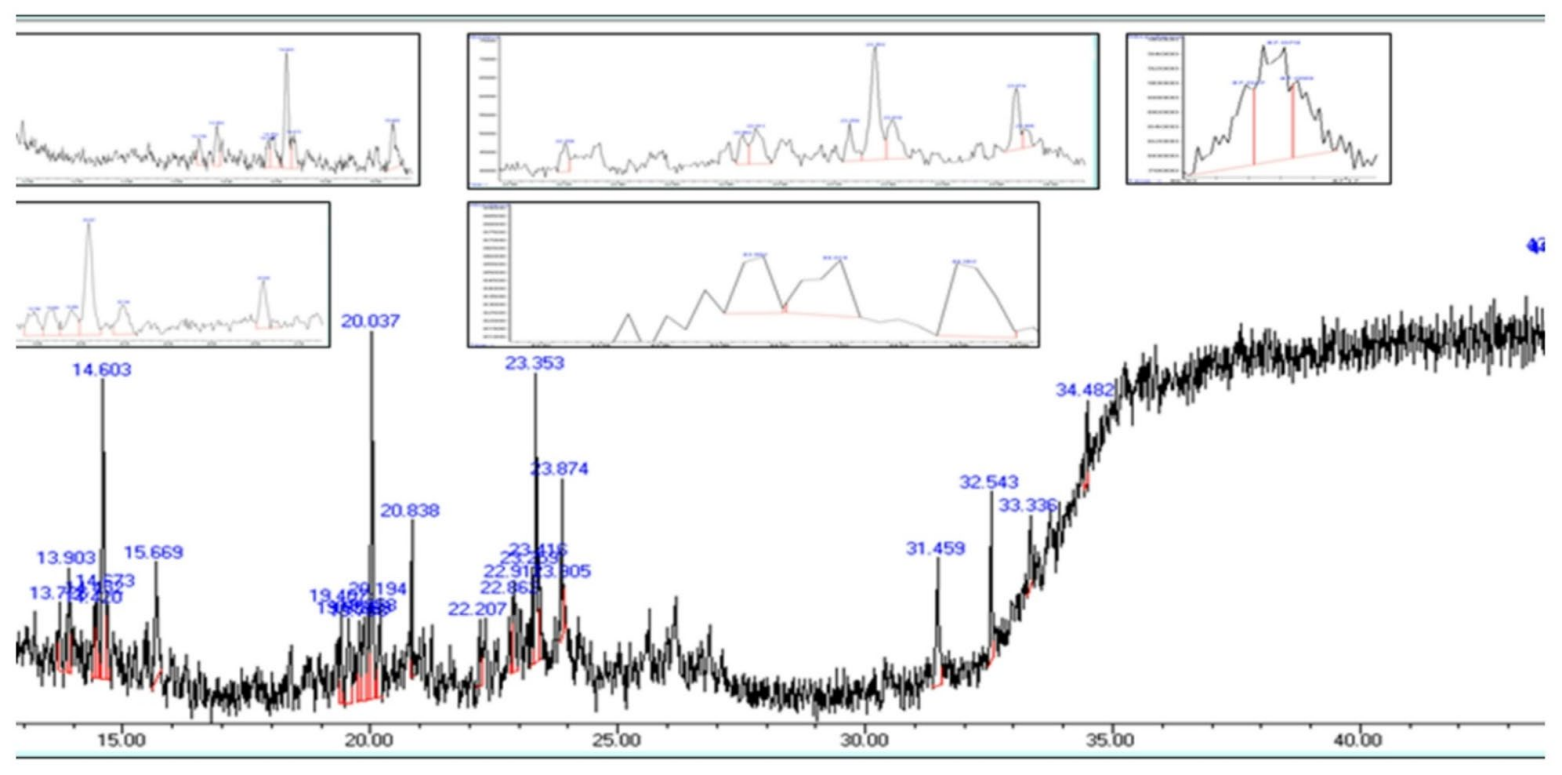
GC MS analysis *Ulva intestinalis* extract.

GC–MS analysis of *Ulva intestinalis* extract identified 36 phytochemical components Fig. 2. Among these, eicosane, pentadecane, tetramethylpentadecane, and 2-methyldecane were the major constituents, representing the highest relative abundances. Several of these compounds have been previously reported to exhibit biological activities, including antimicrobial effects. Compound identification was performed using the NIST/Wiley chemical library to ensure accuracy and reproducibility of the results. Detailed GC–MS spectra and full compound lists are provided in Supplementary as shown in Table 2.

**Table 2:** Bioactive compounds identified in the methanolic extract of *Ulva intestinalis* by GC–MS analysis, along with their respective retention times (RT) and relative peak areas (%).

Peak	Name	*R*. Time	Area%	Molecular formula
1	**1**,**32-Dibromodotriacontane**	**22.913**	**2.4%**	**C**_**32**_ **H**_**64**_ **Br**_**2**_
2	**11**,**20-Didecyltriacontane**	**13.902**	**2.5%**	**C**_**50**_ **H**_**102**_
3	**17-Hexadecyltetratriacontane**	**19.556**	**3.4%**	**C**_**50**_ **H**_**102**_
4	**17-n-Hexadecyltetratriacontane**	**19.407**	**2.4%**	**C**_**50**_ **H**_**102**_
5	**1-Amino-3-(3**,**5-Diiodo-2-Methoxy-Phenyl)-Benzo [4**,**5] Imidazo [1**,**2-** **A] Pyridine-2**,**4-Dicarbonitrile**	**14.672**	**2.2%**	**C**_**19**_ **H**_**10**_ **FN**_**5**_
6	**2-(4**,**5-Dihydro-3-methyl-5-oxo-1-phenyl-4-pyrazolyl)−5-nitrobenzoic acid**	**23.903**	**0.8%**	**C**_**17**_ **H**_**13**_ **N**_**5**_ **O**_**5**_
7	**2**,**2’-Dichloroacetamide**	**11.367**	**1.1%**	**C**_**2**_ **H**_**3**_ **C**_**l2**_ **NO**
8	**2**,**3-Dimethyl-4-Methoxybutanal**	**14.452**	**2.7%**	**C**_**6**_ **H**_**12**_ **O**
9	**2**,**4-Dimethylbenzo[h]quinoline**	**34.479**	**1.5%**	**C**_**15**_ **H**_**13**_ **N**
10	**2**,**6**,**10**,**14-Tetramethyl pentadecane**	**23.353**	**5.9%**	**C**_**19**_ **H**_**40**_
11	**2**,**6-Dichloro-3-phenyl-pyridin**	**15.668**	**3.9%**	**C**_**11**_ **H**_**7**_ **C**_**l2**_ **N**
12	**20**,**25-Dihydroxyaflavinine**	**32.545**	**3.9%**	**C**_**15**_ **H**_**10**_ **O**_**4**_
13	**2-Amino-3-(2-Amino-2-Carboxy-Ethyldisulfanyl)-Propionic Acid**	**14.420**	**1.4%**	**C**_**12**_ **H**_**24**_ **N**_**4**_ **O**_**8**_ **S3**
14	**2 h-Pyrrol-2-One**,**4-Ethyl-5-[[2-[5-[(3-Ethyl-1**,**5-Dihydro-4-Methyl-5-Oxo-2 h-Pyrrol-2-Ylidene) Methyl]−3**,**4-Dimethyl-2 h-Pyrrol-2-Ylidene]−3**,**4-Dimethyl-2 h-Pyrrol-5-Yl] Methylene]−1**,**5-Dihydro-3-Methyl-**,** (E**,** Z**,** Z)-**	**11.244**	**1.2%**	**C**_**22**_ **H**_**24**_ **N**_**2**_ **O**
15	**2-Methyldecane**	**14.601**	**7.5%**	**C**_**11**_ **H**_**24**_
16	**3-(3-Oxo-3 h-Benzo[F]Chromen-2-Yl)−2**,**4(1 h**,**3 h)-Quinolinedione**	**22.861**	**1.3%**	**C**_**14**_ **H**_**8**_ **O**_**4**_
17	**3-(Trans-Pent-4-Enyl)−5**,**5-Dimethylimidazolidine-2**,**4-Dione**	**31.458**	**5.1%**	**C**_**7**_ **H**_**12**_ **N**_**2**_ **O**_**2**_
18	**4**,**5. Alpha. -epoxy-3-methoxy-17-methyl-7. alpha. -(4-phenyl-1**,**3-butadienyl)−6. beta.**,**7. beta. -(oxymet)**	**47.08**	**3.9%**	**C**_**26**_ **H**_**27**_ **NO8**
19	**5-(2-Oxohexahydro-1 h-Thieno[3**,**4-D] Imidazol-4-Yl) Pentanamide**	**19.957**	**2.6%**	**C**_**10**_ **H**_**17**_ **N**_**3**_ **O**_**2**_ **S**
20	**5-Methyloctadecane**	**11.451**	**1.9%**	**C**_**19**_ **H**_**40**_
21	**6-(1-Methylhydrazino) isocytosine hemihydrate**	**43.994**	**3.3%**	**CH**_**6**_ **N**_**2**_
22	**7-Ethyl-6-methyl-5-methylthiopyrazolo[1**,**5-a] pyrimidine**	**44.065**	**1.7%**	**C**_**10**_ **H**_**11**_ **N**_**3**_ **O**_**3**_
23	**9-Octylheptadecane**	**20.837**	**3.3%**	**C**_**25**_ **H**_**52**_
24	**Amphetamine-3-Methyl**	**22.208**	**1.6%**	**C**_**10**_ **H**_**15**_ **N**
25	**Bisphenol**,** bis(tert-butyldimethylsilyl) ether**	**47.099**	**1.9%**	**C**_**27**_ **H**_**44**_ **O**_**2**_ **Si**_**2**_
26	**Cis-Diethyl Ester Of N**,** N’-Dinitro-1**,**2-Cyclohexanedicarbamic Acid**	**33.334**	**1.9%**	**C**_**12**_ **H**_**20**_ **O**_**4**_
27	**Cis-Diethyl Ester Of N**,** N’-Dinitro-1**,**2-Cyclohexanedicarbamic Acid**	**19.789**	**2.6%**	**C**_**12**_ **H**_**20**_ **O**_**4**_
28	**Decane**	**20.196**	**2.9%**	**C**_**10**_ **H**_**22**_
29	**Ethane**,** 1-(4**,**4**,**4-trifluoro-1**,**3-dithiobutyl)−2-(3**,**3**,**3-trifluor**	**44.02**	**1.9%**	**C**_**5**_ **H6F**_**6**_ **S**_**4**_
30	**Heneicosane**	**23.256**	**1.4%**	**C**_**21**_ **H**_**44**_
31	**Hentriacontane**	**23.877**	**2.4%**	**C**_**31**_ **H**_**64**_
32	**Eicosane**	**23.418**	**2.7%**	**C**_**20**_ **H**_**42**_
33	**N-(Cyclohexylcarbonyl)−4-Morpholinecarboxamide**	**13.728**	**1.5%**	**C**_**12**_ **H**_**20**_ **N**_**2**_ **O**_**3**_
34	**N-ethyl-1**,**3-dithioisoindoline**	**47.028**	**2.4%**	**C**_**10**_ **H**_**13**_ **NS**_**2**_
35	**Octadecane**	**19.866**	**2.5%**	**C**_**18**_ **H**_**38**_
36	**Pentadecane**	**20.034**	**8.1%**	**C**_**15**_ **H**_**32**_

### Fourier transform-infrared (FTIR) spectroscopic analysis

FTIR analysis of *Ulva intestinalis* extract revealed various functional groups indicative of bioactive compounds. Prominent bands corresponded to hydroxyl and amino groups (O–H/N–H), carbonyl groups (C = O), sulfate groups (S = O), and carbohydrate moieties, suggesting the presence of amino acids, polysaccharides, esters, and other bioactive molecules. These functional groups may contribute to the observed antimicrobial activity. Detailed spectra and peak assignments are presented in Supplementary Fig. 3.

**Fig. 3:**
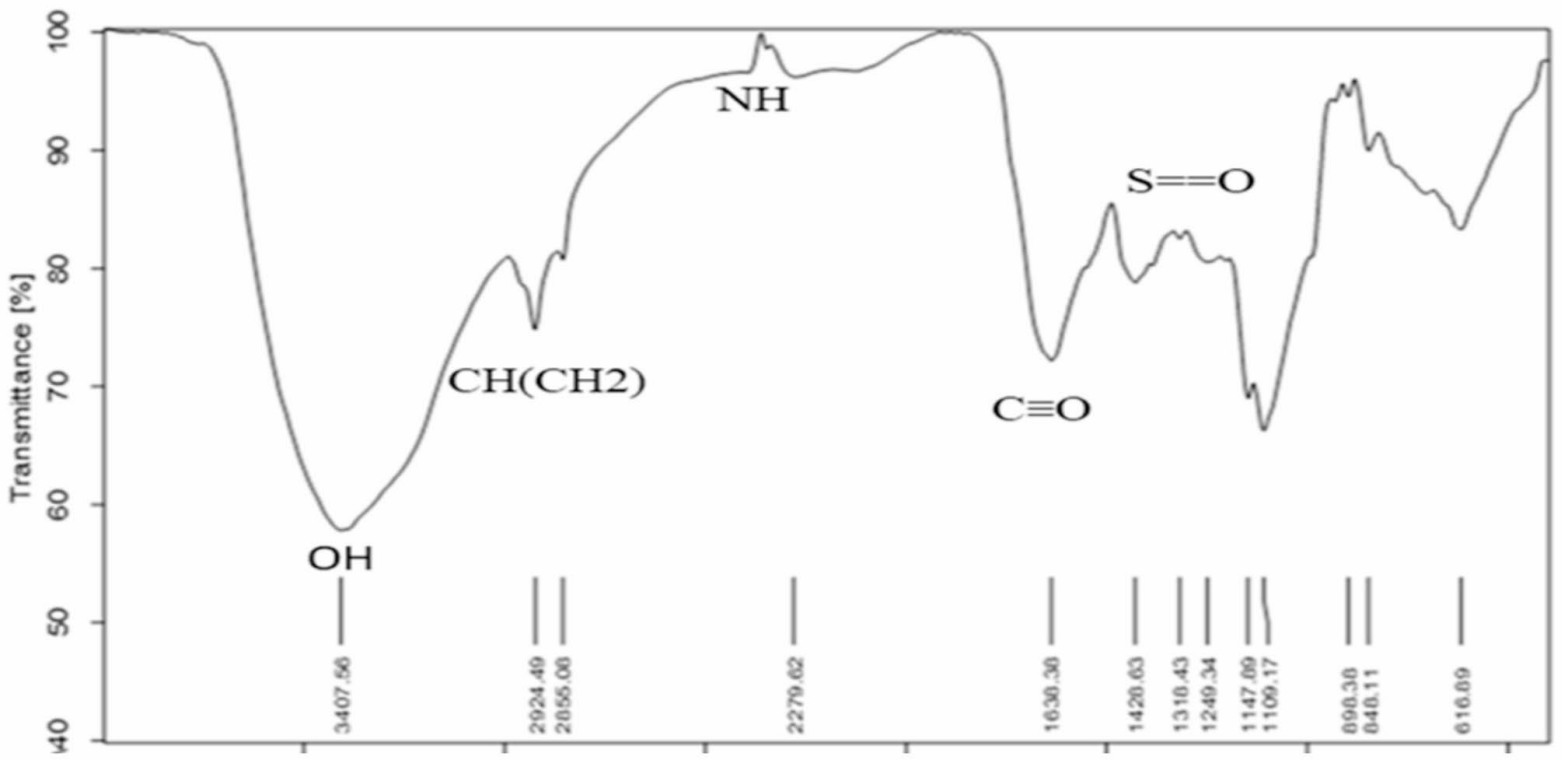
Fourier transform infrared spectrum of *Ulva intestinalis.*

### Docking investigation of significant targets from ***staphylococcus aureus***

**Fig. 4:**
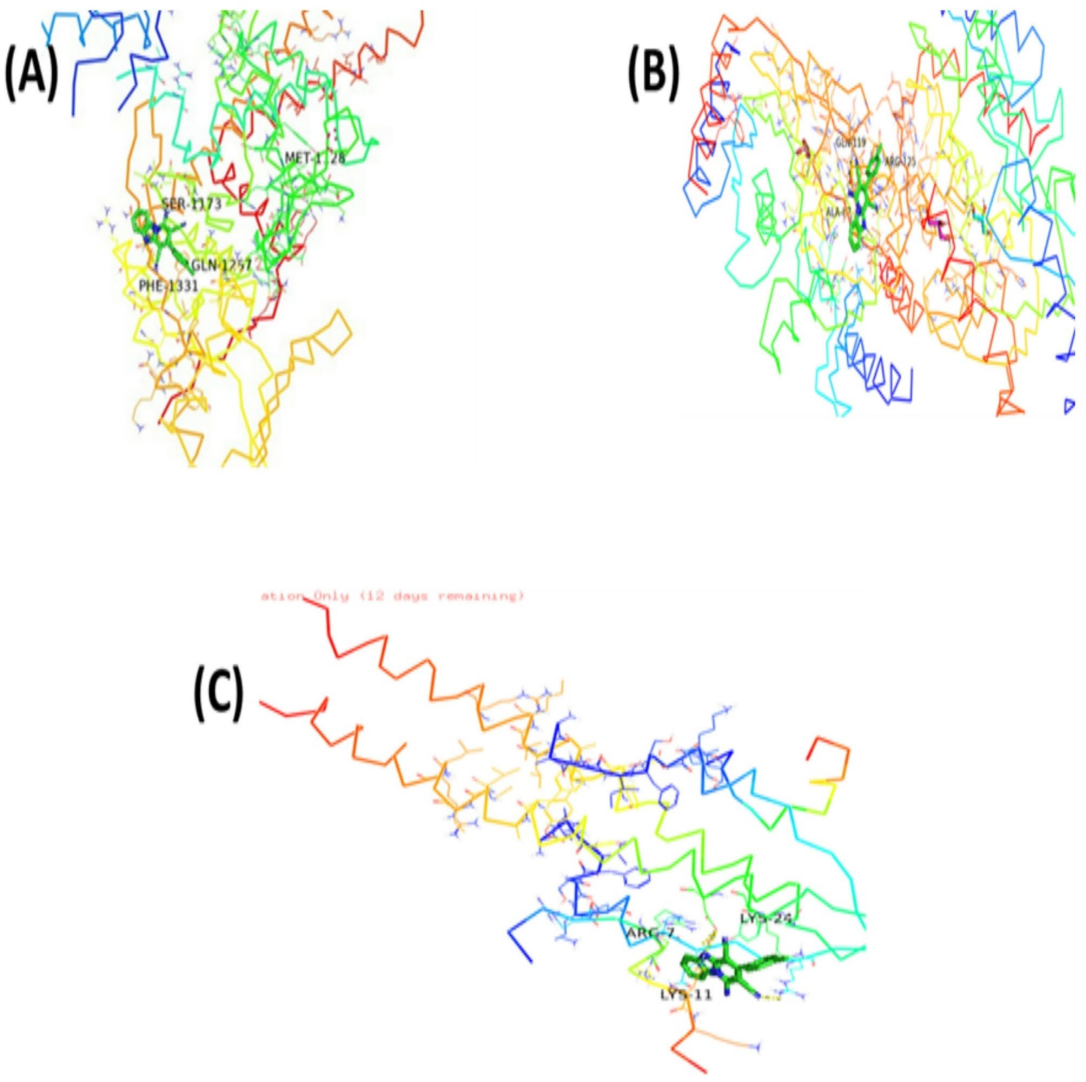
Docked pose of the ligand (1-amino-3-(3,5-diiodo-2-methoxyphenyl)-benzo^4,5^ imidazole[1,2-a] pyridine-2,4-dicarbonitrile) (**A**) DNA gyrase (**B**) RNA polymerase (**C**) penicillin-binding protein 2a (PBP2a).

**Fig. 5:**
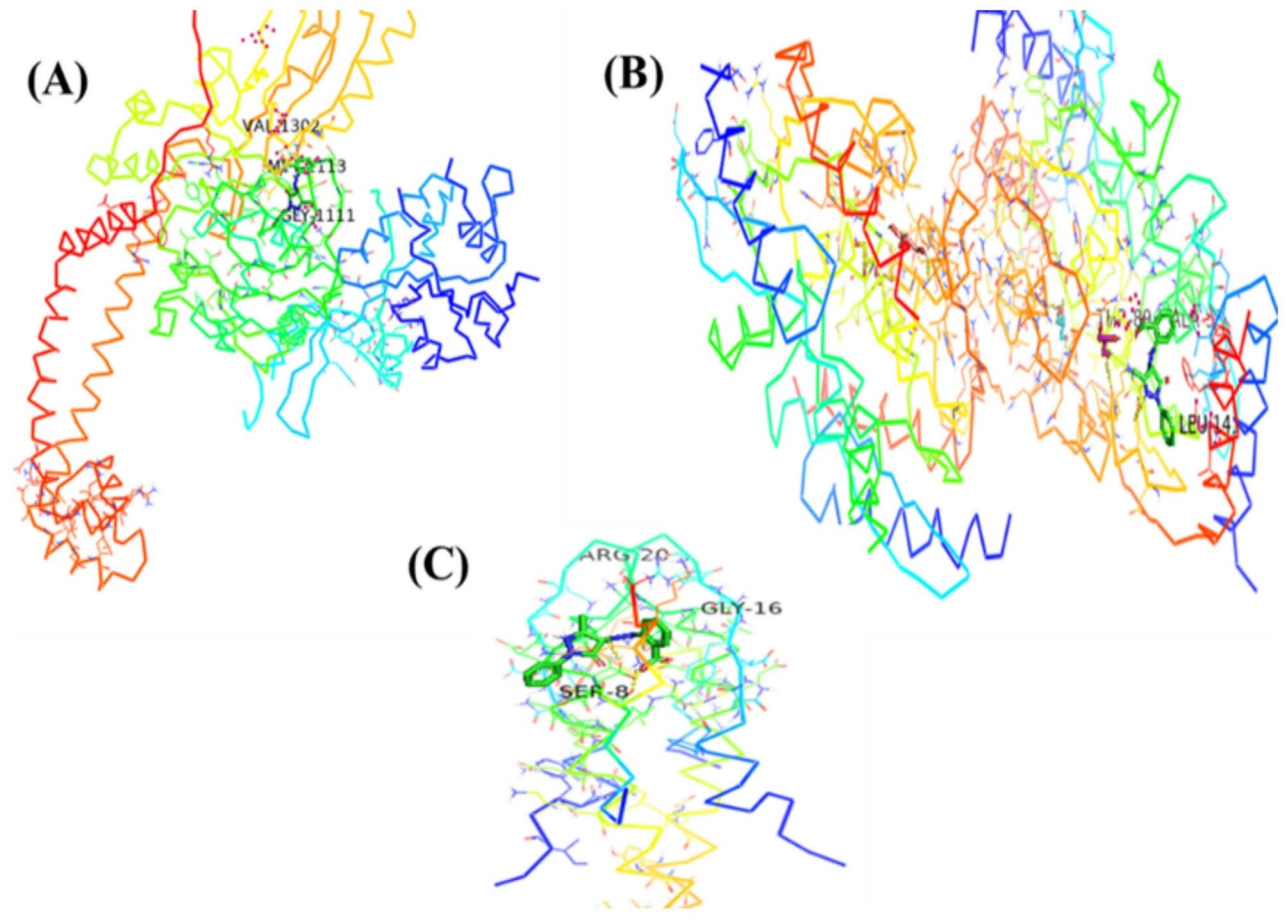
Docked pose of the ligand (4,5-dihydro-3-methyl-5-oxo-1-phenyl-4-pyrazolyl)−5-nitrobenzoic (**A**) DNA gyrase (**B**) RNA polymerase (**C**) penicillin-binding protein 2a (PBP2a).

Molecular docking studies were performed using crystal structures of *Staphylococcus aureus* DNA gyrase (PDB ID: 6Z1A), RNA polymerase (PDB ID: 8 × 6 F), and penicillin-binding protein 2a (PDB ID: 5M18), retrieved from the RCSB Protein Data Bank, selected due to their established roles in bacterial replication, transcription, and β-lactam resistance. The docking scores and key interaction parameters of the identified bioactive ligands against these targets are summarized in Table 3. The ligand 1-amino-3-(3,5-diiodo-2-methoxyphenyl)-benzo^4,5^imidazo[1,2-a]pyridine-2,4-dicarbonitrile exhibited the highest binding affinity to DNA gyrase (–7.6 kcal/mol), forming multiple hydrogen bonds and halogen interactions with catalytically significant residues. The iodine atoms contributed to halogen bonding, which may enhance selectivity, aligning with the known importance of halogen bonds in inhibitor potency^10^. This ligand also showed favorable binding to RNA polymerase (–8.2 kcal/mol) and PBP2a (–7.7 kcal/mol) through hydrogen bonds, hydrophobic contacts, and van der Waals interactions, suggesting potential interactions with key active-site residues (Fig. 4). Similarly, (4,5-dihydro-3-methyl-5-oxo-1-phenyl-4-pyrazolyl)−5-nitrobenzoic acid derived from *Ulva intestinalis* demonstrated the highest affinity for PBP2a (–7.2 kcal/mol) via persistent hydrogen bonds and hydrophobic interactions within the β-lactam-binding pocket. It also formed stabilizing interactions with DNA gyrase (–6.7 kcal/mol) and RNA polymerase (–7.8 kcal/mol), including hydrogen bonds and hydrophobic contacts, indicating potential binding to residues involved in enzymatic activity (Fig. 5). In both ligands, docking poses revealed stable conformations supported by networks of hydrogen bonds, hydrophobic interactions, and halogen bonds (for the heterocyclic ligand). These results highlight possible modes of interaction with critical bacterial targets, although experimental validation is required to confirm any biological activity.

**Table 3:** Molecular Docking results of bioactive ligands against *Staphylococcus aureus* targets.

Protein Target	Ligand	Binding Affinity (kcal/mol)	Key Interactions
**DNA gyrase**	**1-amino-3-(3,5-diiodo-2-methoxyphenyl)-benzo[4,5]imidazole[1,2-a]pyridine-2,4-dicarbonitrile**	**–7.6**	**H-bonds with catalytic residues, halogen bonding (I atoms), hydrophobic contacts**
**DNA gyrase**	**(4,5-dihydro-3-methyl-5-oxo-1-phenyl-4-pyrazolyl)−5-nitrobenzoic acid**	**–6.8**	**H-bonds with active site residues, hydrophobic contacts**
**RNA polymerase**	**1-amino-3-(3**,**5-diiodo-2-methoxyphenyl)-benzo[4**,**5]imidazole[1**,**2-a]pyridine-2**,**4-dicarbonitrile**	**–8.2**	**H-bonds and hydrophobic interactions within catalytic cleft**
**RNA polymerase**	**(4**,**5-dihydro-3-methyl-5-oxo-1-phenyl-4-pyrazolyl)−5-nitrobenzoic acid**	**–7.5**	**H-bonds and hydrophobic interactions**
**PBP2a**	**1-amino-3-(3**,**5-diiodo-2-methoxyphenyl)-benzo[4**,**5]imidazole[1**,**2-a]pyridine-2**,**4-dicarbonitrile**	**–7.7**	**H-bonds and van der Waals contacts in β-lactam-binding region**
**PBP2a**	**(4**,**5-dihydro-3-methyl-5-oxo-1-phenyl-4-pyrazolyl)−5-nitrobenzoic acid**	**–7.0**	**H-bonds and hydrophobic contacts**

## Discussion

The findings of this study demonstrate that *Ulva intestinalis* extracts exhibit strong antibacterial and antibiofilm activities against *Staphylococcus aureus*, including antibiotic-resistant clinical isolates. Compared with previously reported seaweed-derived antimicrobials, the extracts showed remarkable activity against both planktonic growth and biofilm formation, highlighting their potential pharmacological relevance. Among the tested extracts, methanolic and ethanolic fractions exhibited the highest antibacterial activity, with inhibition zones of 33.03 ± 0.24 mm and 35.03 ± 0.04 mm, respectively, exceeding that of gentamicin under identical experimental conditions. These results are consistent with previous reports indicating that polar solvents efficiently extract phenolic and flavonoid compounds from green algae, which are responsible for their antimicrobial activity^11,12^. Acetone extracts exhibited comparatively lower yet measurable antibacterial activity, aligning with prior observations that less polar solvents can still yield antimicrobial constituents effective against Gram-positive bacteria^13^.

All extracts also exhibited notable antibiofilm formation inhibitory effects, indicating interference with bacterial adhesion and extracellular matrix production during the early stages of biofilm development. The observed inhibition (91.26% at 200 µg/mL) aligns with previous studies reporting that ulvan-rich fractions of *Ulva* spp. and extracts of *U. lactuca* effectively reduce biofilm formation^14–19^. Seasonal and environmental factors may also influence the metabolite composition of *U. intestinalis*; for instance, samples collected during winter were previously found to contain higher levels of nonpolar compounds, which may enhance their antibacterial potential^20^. These findings highlight the importance of solvent polarity, seasonal variability, and the presence of sulfated polysaccharides such as ulvan in shaping both antibacterial and antibiofilm formation inhibitory activities^21,22^. Although the concentration used in the antibiofilm assay (200 µg/mL) overlaps with the antibacterial range, it was selected after several preliminary trials to ensure reproducible and measurable inhibition of biofilm formation. Therefore, the observed antibiofilm effects are likely attributed to a combination of bacteriostatic and anti-adhesion mechanisms. Future studies will aim to determine the minimum inhibitory concentration (MIC) and evaluate antibiofilm formation inhibition at sub-MIC levels to distinguish these effects more precisely. GC–MS analysis of *U. intestinalis* extracts identified several biologically active compounds, including eicosane (6.99%), pentadecane (6.23%), and tetramethylpentadecane (5.97%), all of which have been previously reported to possess antibacterial or antifungal properties^23^. The predominance of hydrocarbons and fatty acid derivatives suggests their synergistic contribution to the overall antibacterial effect. However, the absence of MS/MS fragmentation analysis limits detailed structural elucidation; this will be addressed in future work. FT–IR spectral analysis supported the presence of functional groups characteristic of phenolics, amines, sulfated polysaccharides, and fatty acids, consistent with previously described seaweed metabolites^24–35^. The detection of sulfate esters and polysaccharide peaks confirms the biochemical complexity underlying the antimicrobial and antibiofilm effects of *U. intestinalis*. It is important to note that these analyses were performed on the crude extract; therefore, FT–IR analysis of purified individual compounds in future studies would provide more accurate structural characterization. Additionally, FT–IR can be employed to examine the effect of *U. intestinalis* extracts on extracellular polymeric substances (EPS) within biofilms, as demonstrated in related studies, to further elucidate the mechanisms underlying antibiofilm activity. Molecular docking analysis provided insight into potential interactions between bioactive compounds from *U*. *intestinalis* and essential *S. aureus* targets, including PBP2a, RNA polymerase, and DNA gyrase, consistent with previous studies using phytochemicals to target bacterial quorum sensing and protein interactions^36^. Both ligands exhibited favorable binding affinities with these proteins, suggesting possible interactions with catalytically important residues^37,38^. The synthetic ligand 1-amino-3-(3,5-diiodo-2-methoxyphenyl)-benzo^4,5^imidazo[1,2-a]pyridine-2,4-dicarbonitrile displayed the highest affinity for DNA gyrase (–7.6 kcal/mol), while the natural compound (4,5-dihydro-3-methyl-5-oxo-1-phenyl-4-pyrazolyl)−5-nitrobenzoic acid showed strong binding to PBP2a (–7.2 kcal/mol) and RNA polymerase (–7.8 kcal/mol) as shown in Table 3. The stability of these interactions, supported by hydrogen bonds, hydrophobic contacts, and halogen bonding, indicates potential multitarget binding patterns consistent with other marine-derived antibacterials^39–45^. These findings suggest that *U. intestinalis* metabolites may interact with multiple bacterial targets, though further experimental validation is required to confirm their antimicrobial and antibiofilm efficacy. Overall, the present results are consistent with previous studies on *Ulva* species but extend existing knowledge by demonstrating that *U. intestinalis* extracts possess potent dual antibacterial and antibiofilm activities against *S. aureus*, including antibiotic-resistant strains. This highlights *U. intestinalis* as a promising natural source of anti-virulence agents that could serve as complementary or alternative strategies to conventional antibiotics, potentially reducing the risk of antimicrobial resistance development.

## Conclusion

The present study demonstrates that *Ulva intestinalis* extracts possess potent antibacterial and biofilm formation inhibitory activities against *Staphylococcus aureus*. The observed bioactivity is likely associated with the presence of diverse secondary metabolites, as revealed by FTIR and GC–MS analyses, which identified phenolic compounds, fatty acids, and sulfated polysaccharides known for their antimicrobial potential. These findings highlight *U. intestinalis* as a promising natural source of bioactive compounds capable of interfering with bacterial growth and the initial stages of biofilm development. However, since only one *S. aureus* strain was tested, the results should be considered preliminary. Future studies should include multiple clinical isolates, particularly antibiotic-resistant strains, and focus on bioassay-guided purification, structural elucidation using spectroscopic methods, and in vivo validation to confirm safety and efficacy. Overall, this study provides a scientific basis for the potential use of *U. intestinalis* as an eco-friendly antimicrobial and biofilm formation-inhibiting agent, contributing to the search for sustainable alternatives to conventional antibiotics.

## Materials and methods

### Sample collection and preparation

The green seaweed *Ulva intestinalis* was collected from Alexandria, Egypt (31°21′46″N, 29°88′49.4″E) in March. The algal biomass was hand-picked, thoroughly washed several times with seawater to remove epiphytes, debris, and sand particles, and then rinsed with distilled water. The cleaned biomass was air-dried and subsequently ground into a fine powder using an electric blender. Taxonomic identification of the seaweed was performed according to the references of Guiry & Guiry, Kanaan & Belous, and Aleem^46–48^.

### Seaweed extracts preparation

Modified versions of previously published methods^49,50^ were employed to prepare three different extracts of *Ulva intestinalis*. Briefly, 10 g of dried seaweed powder was extracted with 150 mL of each solvent (methanol, ethanol, and acetone) for 24 h at 50 °C using a Soxhlet extractor. The resulting extracts were then concentrated by evaporation at 40 °C to remove all solvents completely. The dried residues were subsequently reconstituted and used in all antibacterial assays to avoid any solvent-related toxicity. Methanol, ethanol, and acetone were chosen due to their proven efficiency in extracting a broad spectrum of bioactive compounds from marine algae. Finally, the crude extracts were collected and stored in sealed vials at − 20 °C to preserve their bioactive components until further use.

### Bacterial strain

The bacterial strain used in this study was a *Staphylococcus aureus* clinical isolate obtained from the Microbiology Laboratory, Faculty of Medicine, Sohag University Hospital. The isolate was confirmed as methicillin-resistant *S. aureus* (MRSA) based on its antibiotic susceptibility profile. Further identification was verified through Gram staining and standard biochemical assays, including catalase, coagulase, and mannitol fermentation tests, to ensure the accuracy and purity of the isolate^48,51^.

### Screening for antibacterial activity

The antibacterial activity of *Ulva intestinalis* extracts (methanolic, ethanolic, and acetone) was evaluated using the agar well diffusion method^52^. Briefly, 15 mL of sterile nutrient agar medium was poured into Petri dishes and allowed to solidify. Then, 1 mL of a bacterial suspension of *Staphylococcus aureus* (clinical isolate obtained from Sohag University Hospital, Egypt), adjusted to 0.5 McFarland standard (≈ 1.5 × 10⁸ CFU/mL), was evenly spread on the agar surface using a sterile swab. Wells (6 mm in diameter) were created using a sterile stainless-steel cork borer, and 100 µL of each reconstituted dried extract was added to the wells. Gentamicin (50 µg/100 µL; Egyptian International Pharmaceutical Industries Company, EIPICO; commercial name “EPIGENT”) was used as a positive control, and DMSO served as a negative control to rule out solvent effects. All experiments were performed in triplicate. The plates were incubated at 37 °C for 24 h under aerobic conditions, and antibacterial activity was determined by measuring the diameter of the inhibition zones (mm). Data are presented as mean ± standard deviation. Statistical analysis was performed using one-way ANOVA followed by Tukey’s post hoc test to determine significant differences between treatments (*p* < 0.05). This procedure ensured that the observed antibacterial activity was solely attributable to *U. intestinalis* extracts.

### Antibiofilm assay

The antibiofilm potential of the methanolic extract of *Ulva intestinalis* against *Staphylococcus aureus* was evaluated using the 96-well microtiter plate method^53^. Briefly, bacterial cultures were grown overnight in nutrient broth at 37 °C, adjusted to 0.5 McFarland standard (≈ 1.5 × 10⁸ CFU/mL), and diluted to achieve a final inoculum size of approximately 1 × 10⁶ CFU/mL in sterile tryptic soy broth (TSB) supplemented with 1% glucose to promote biofilm formation. The bacterial suspension was incubated with different concentrations of the algal extract (50, 100, and 200 µg/mL) in sterile flat-bottom 96-well plates at 37 °C for 24 h under static conditions. After incubation, planktonic cells were carefully removed, and wells were gently washed three times with 200 µL of sterile phosphate-buffered saline (PBS, pH 7.4) to remove non-adherent bacteria. Plates were air-dried for 15–20 min and stained with 0.1% (w/v) crystal violet for 15 min. Excess stain was rinsed off with distilled water, and the bound dye was solubilized with 95% ethanol. Absorbance was measured at 630 nm using a microplate reader. The percentage of biofilm inhibition was calculated relative to the untreated control using the formula: % biofilm inhibition = 1 – (OD630 of cells treated with seaweeds/OD630of non-treated control) ×100. The lowest concentration that effectively inhibited biofilm formation was determined. All experiments were conducted in triplicate, and results are expressed as mean ± standard deviation. Statistical analysis was carried out using one-way ANOVA followed by Tukey’s post hoc test (*p* < 0.05).

### Gas chromatography–mass spectrometry (GC–MS)

Gas chromatography–mass spectrometry (GC–MS) combines the separation capabilities of gas chromatography with the identification power of mass spectrometry to characterize compounds present in a sample^54^. Chromatographic separation was performed using an Assiut University GC–MS system (7890 − 5975) equipped with an HP-5ms column (30 m × 0.25 mm × 0.25 μm). The GC oven temperature was programmed to rise from 40 °C to 280 °C at a rate of 10 °C/min, with a hold at 150 °C for 6 min. Helium was used as the carrier gas at 1 mL/min. Prior to analysis, the methanolic extract was filtered through a 0.45 μm membrane to remove particulate matter, ensuring consistent injection and minimizing artifacts. Compound identification was based on comparison with previously reported GC–MS data for *Ulva intestinalis* and related seaweed extracts. No authentic chemical standards were used in this study.

### Fourier transform infrared spectroscopy (FTIR)

FT-IR spectra of the *Ulva intestinalis* methanolic extract were recorded using a JASCO FT-IR spectrophotometer (FT-IR-6100; JASCO, Tokyo, Japan) in the spectral region of 4000–400 cm⁻¹ at a resolution of 2 cm⁻¹. The crude extract was dried under controlled conditions and thoroughly mixed with KBr to form uniform pellets, ensuring reproducibility and minimizing potential artifacts. This technique provides rapid sampling, excellent reproducibility, and allows a general overview of functional groups present in the extract, rather than analysis of purified individual compounds^55^.

### Docking analysis

**Fig. 6:**
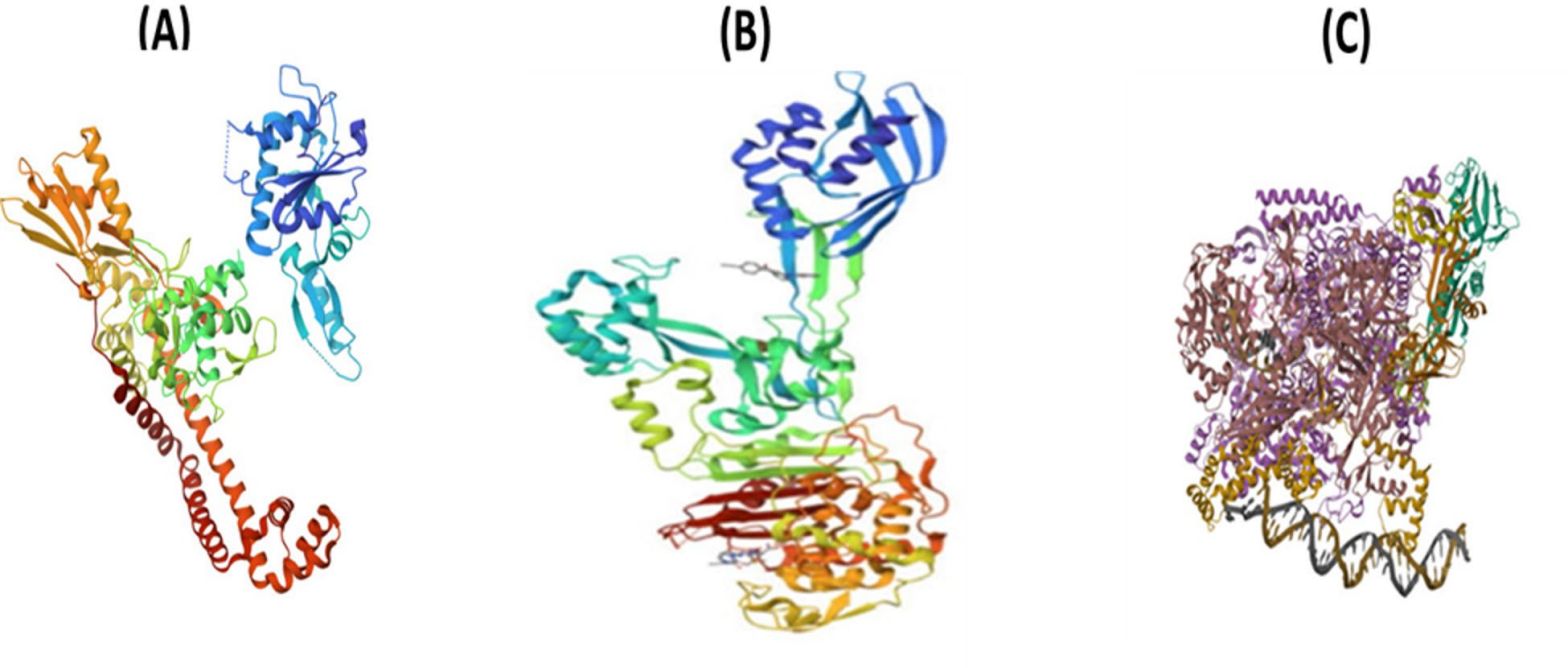
3D docking poses of ligands with (**A**) DNA gyrase, (**B**) PBP2 protein, and (**C**) RNA polymerase.

**Fig. 7:**
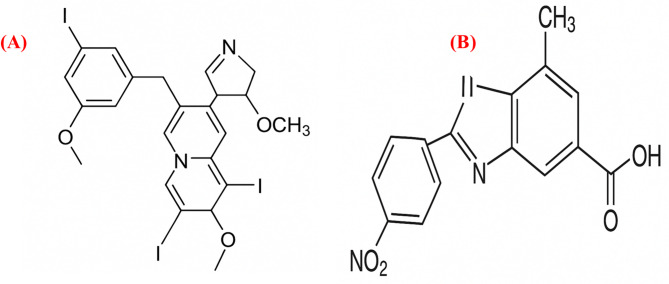
Chemical structure (**A**) 1-amino-3-(3,5-diiodo-2-methoxyphenyl)-benzo^4,5^ imidazole[1,2-a] pyridine-2,4-dicarbonitrile, (**B**) (4,5-dihydro-3-methyl-5-oxo-1-phenyl-4-pyrazolyl)−5-nitrobenzoic acid.

 This study investigated the binding interactions between two bioactive ligands and three bacterial proteins that play essential roles in the pathogenicity and antibiotic resistance of *Staphylococcus aureus*. DNA gyrase (PDB ID: 6Z1A), RNA polymerase (PDB ID: 8 × 6 F), and penicillin-binding protein 2a (PDB ID: 5M18), retrieved from the RCSB Protein Data Bank. (Fig. 6) were chosen based on their critical roles in bacterial DNA replication and β-lactam resistance^37,38^. The ligands tested were 1-amino-3-(3,5-diiodo-2-methoxyphenyl)-benzo^4,5^ imidazole[1,2-a]pyridine-2,4-dicarbonitrile, a heterocyclic compound with reported antimicrobial activity, and (4,5-dihydro-3-methyl-5-oxo-1-phenyl-4-pyrazolyl)−5-nitrobenzoic acid (Fig. 7), a nitro-substituted benzoic acid isolated from the marine alga *Ulva intestinalis*. The docking study focused on these two ligands due to their reported antimicrobial activity and relevance to the selected *S. aureus* targets, despite the presence of other compounds. These ligands were selected as major bioactive compounds identified through GC–MS analysis of the algal extracts. Ligand structures were subjected to energy minimization and geometry optimization using the Universal Force Field (UFF) implemented in PyRx v0.8^56^. The minimized structures were saved in PDBQT format for docking analysis. Docking simulations were performed using AutoDock Vina implemented in PyRx v0.8. For each protein, the docking grid was centered on the active site residues identified from literature, ensuring that the binding pocket was fully covered. The exhaustiveness parameter was set to 8 to enhance conformational sampling^57^. Binding affinities (measured in kcal/mol) and docking poses were recorded for each ligand–protein complex. The best docking poses were selected based on the lowest binding free energy and the number of favorable non-covalent interactions, including hydrogen bonds, hydrophobic contacts, and halogen bonding. Visualization and interaction analysis were conducted using PyMOL, with identification of the key amino acid residues involved in ligand binding^58^. Although molecular docking provides predictive insights into potential binding interactions, these results serve as a mechanistic complement to the in vitro antibacterial and antibiofilm assays rather than direct experimental proof. The predicted interactions, suggest possible modes of inhibition of bacterial replication and resistance pathways, supporting the observed bioactivity of *U. intestinalis* extracts.

### Statistics analysis

All data were expressed as mean ± SD of triplicates. Statistical analysis was performed using one-way ANOVA followed by Tukey’s post hoc test, with *p* < 0.05 considered statistically significant.

## Data Availability

The data supporting the findings of this study are available from the corresponding author upon reasonable request.
